# Supplementary material for: Human uniqueness? Life history diversity among small-scale societies and chimpanzees
Source: PLoS One. 2021 Feb 22;16(2):e0239170. doi: 10.1371/journal.pone.0239170 (PMC7899333; doi:10.1371/journal.pone.0239170)
Supplement: S1 Data — (DOCX) [file pone.0239170.s001.docx]

**Supporting Information for Davison and Gurven “**Human uniqueness? Life history diversity among small-scale societies and chimpanzees**”**

**Table of Contents**

**S1 Text: Supporting Methods**

1. Population metadata and ethnographic details
2. Mortality smoothed with a Siler model
3. Fertility smoothed with Loess regression
4. Matrix model construction and emergent life history traits
5. Force of selection: vital rate sensitivities and elasticities
6. Life table response experiments (LTRE): vital rate fitness contributions

**S2 Text: Supporting Results**

1. Population growth and decline
2. Mortality and fertility patterns

**S3 Text: Supporting References**

**Supporting Tables**

S1 Table. Study populations and metadata.

S2 Table. Age-specific survival probabilities (*p_x_*) for study populations

S3 Table. Age-specific fertility rates (*m_x_*) for study populations

S4 Table. Mann-Whitney-Cox U-statistics and P-values for life history differences among populations

S5 Table. Mann-Whitney-Cox U-statistics and P-values for within-population differences among measures

**Supporting Figures**

Figure S1. Fertility smoothing comparison (raw vs. smoothed)

Figure S2. Tempo of fertility comparision (ages at first and last birth)

Figure S3. Mortality and fertility rates for individual populations

Figure S4. Vital rate elasticities for individual populations

Figure S5. LTRE contributions among small-scale societies

Figure S6. LTRE contributions among chimpanzees

Figure S7. Net LTRE contributions among small-scale societies and chimpanzees

Figure S8. LTRE effect magnitudes among small-scale societies and chimpanzees

**Supporting Information for Davison and Gurven “**Human uniqueness? Life history diversity among small-scale societies and chimpanzees**”**

**S1 File: Supporting Methods**

**1. Ethnographic information on human study populations**

Contemporary hunter-gatherers have been affected by global socioeconomic forces and are not living replicas of our Stone Age ancestors. Each group has been exposed to a particular set of historical, ecological, and political conditions, and extant groups occupy only a small subset of the environments that foragers occupied in the past. Thus, even without the variable impact of infectious diseases and modernization, no single group can accurately represent all modern foragers or pristine foragers typical of our ancestral past (see [*Solway*](#_ENREF_1) *et al., 1990*). Isolation from outsiders, small-scale social structure, and absence of amenities also characterize many incipient horticulturalist populations, many of whom also engage in foraging. Remote populations of forager-horticulturalists therefore merit attention, especially when considering analogues for Holocene domestication during the Neolithic demographic transition. For every population in our study, we examine data from the earliest phase of contact (see Table 1 for population metadata and source references).

### **Hunter-gatherers**

The ethnographic record of hunter-gatherers includes hundreds of cultures, but only fifty or so groups have been studied. The sample of foraging societies presented here does not adequately cover all geographical areas. Only five foraging societies have been explicitly studied using demographic techniques— the Hadza of Tanzania (*Blurton Jones et al., 1992; Blurton Jones et al., 2002; Blurton Jones, 2016*), the Ju/’hoansi !Kung (*Howell, 1979, 2010*), the Ache of Paraguay (*Hill and Hurtado, 1996*), the Agta of Philipines (*Early and Headland, 1998*), and the Hiwi of Venezuela (*Hill et al., 2007*).

Nancy Howell’s **Ju/’hoansi !Kung** study in the Kahalari desert of Botswana and Namibia is one of the first and most impressive demographic accounts of a foraging society. The majority of !Kung have been settled during the last sixty years, and have been rapidly acculturating in close association with nearby Herero and Tswana herders. At the time of study, many of the adults had spent most of their lives foraging, despite ethnohistorical evidence showing interactions with mercantile interests in the 19^th^ century and archaeological evidence suggesting trade with pastoral and agricultural populations. The early !Kung sample we use refers to the time period before the 1950’s when the Bantu influence in the Dobe area was minimal. Later !Kung samples refer to the prospective time of study when the lifeways of the Kung were rapidly changing. At the time of study, there were about 454 people living in the study site.

The **Ache** were full-time, mobile tropical forest hunter-gatherers until the 1970’s. Hill and Hurtado (*Hill and Hurtado, 1996*) separate Ache history into three time periods—a precontact “forest” period of pure foraging with no permanent peaceful interactions with neighboring groups (before 1970, used in this study), a “contact” period (1971-77) where epidemics had a profound influence on the population, and a recent “reservation” period where they live as forager-horticulturalists in relatively permanent settlements (1978-1993). During this latter period, the Ache have had some exposure to health care. The pre-contact Ache period shows marked population increase, due in part to the open niche that was a direct result of high adult mortality among Paraguayan nationals during the Chaco War with Bolivia in the 1930’s. No life table is published for the high mortality contact period which killed many older and young individuals. Hill and Hurtado (*Hill and Hurtado, 1996*) improve on Howell’s methods of age estimation by using averaged informant ranking of age, informant estimates of absolute age differences between people, and polynomial regression of estimated year of birth on age rank. Apart from living individuals, reproductive histories of a large sample of adults built the samples used for mortality analysis. At the time of study, there were roughly 570 Northern Ache.

The **Hadza** in the eastern rift valley of Tanzania were studied in the mid-1980’s by Nicholas Blurton Jones and colleagues. Trading with herders and horticulturalists has been sporadic among Hadza over the past century, and the overall quantity of food coming from horticulturalists varies from 5-10% (*Blurton Jones, 2002*). The Hadza have been exposed to a series of settlement schemes over the past fifty years, but none of these has proven very successful. The 1990’s saw a novel form of outsider intervention in the form of further habitat degradation and “ethno-tourism” (*Blurton Jones, 2002*). Although some Hadza had spent considerable time living in a settlement with access to maize and other agricultural foods, most had not and continue to forage and rely on wild foods. The population was aged using relative age lists, a group of individuals of known ages, and polynomial regression. Two censuses done about fifteen years apart, with an accounting of all deaths during the interim, allowed (*Blurton Jones, 2002*) to construct the life table we use here, and to further show that sporadic access to horticultural foods and other amenities cannot account for the mortality profile. There were roughly 750 Hadza in the study population.

The **Hiwi** are neotropical savanna foragers of Venezuela studied by Kim Hill and Magdalena Hurtado in the late 1980’s (*Hurtado and Hill 1987, 1990*). They were contacted in 1959 when cattle ranchers began encroaching into their territory. Although living in semi-permanent settlements, Hiwi continue to engage in violent conflict with other Hiwi groups. At the time of study, almost the entire diet was wild foods, with 68% of calories coming from meat, and 27% from roots, fruits, and an arboreal legume. The study population contains a total of 781 individuals. Nearby Guahibo-speaking peoples practice agriculture, while the Hiwi inhabited an area poorly suited for agriculture. As among the Hadza, repeated attempts at agriculture by missionaries or government schemes had failed among this group. Mortality information comes from (*Hill et al., 2007*).

The Casiguran **Agta** of the Philippines are Negrito foragers studied by Tom Headland from 1962-1986. They live on a peninsula close to mountainous river areas and the ocean. Of the 9,000 Agta in eastern Luzon territory, the demographic study was focused on the San Ildefonso group of about 200 people (*Early and Headland, 1998*). Although the Luzon area is itself very isolated, Agta have maintained trading relationships with lowland horticulturalists for at least several centuries (*Headland, 1997*). The twentieth century introduced schooling, and brief skirmishes during American and Japanese occupation. Age estimation was achieved through reference to known ages of living people and calendars of dated events. As in the Ache study, the Agta demography is divided into a “forager” period (1950-1965, used in our study), a transitional period of population decline (1966-1980), and a “peasant” phase (1981-1993). These latter phases are marked by guerilla warfare, and subjugation by loggers, miners and colonists.

### **Forager-horticulturalists**

The above five populations comprise the foraging sample because the typology “hunter-gatherer” defines their mode of subsistence, and therefore a lack of reliance on domesticated foods. To the forager sample described above, we add the Yanomamo of Venezuela and Brazil, Tsimane of Bolivia and Gainj of Papua New Guinea.

The Yanomamo and Tsimane are forager-horticulturalist populations in Amazonian South America. Several different Yanomamo studies have been carried out over the past thirty years. Although often construed as hunter-gatherers, **Yanomamo** have practiced slash and burn horticulture of plantains for many generations (*Chagnon, 1968*). They mostly live in small villages of less than fifty people. The effects of the rubber boom and slave trade before the 18^th^ century on Yanomamo were minimal. The Yanomamo remained mostly isolated until missionary contact in the late 1950’s. The most complete demography comes from Early and Peters *(Early and Peters, 2000*) based on prospective studies of eight villages in the Parima Highlands of Brazil. Births and deaths were recorded by missionaries and FUNAI personnel since 1959. The precontact period (1930-56, used in the present study) predates missionary and other outside influence. The contact period (1957-60), “linkage” period (1961-81) and Brazilian period (1982-96) saw increased interaction with miners, Brazilian nationals and infectious disease. Ages during this period were estimated using a chain of average interbirth intervals for people with at least one sibling of known age, and relative age lists in combination with estimated interbirth intervals. These censuses were taken during the 1960’s, and ages were obtained by averaging different researchers’ independent guesses.

The **Tsimane** inhabit tropical forest areas of the Bolivian lowlands, congregating in small villages near large rivers and tributaries. There are roughly 16,000 Tsimane living in dispersed settlements in the Beni region. The Tsimane have had sporadic contact with Jesuit missionaries since before the 18^th^ century, although were never successfully converted or settled. Evangelical and Catholic missionaries set up missions in the early 1950’s, and later trained some Tsimane to become teachers in the more accessible villages. However, the daily influence of missionaries is minimal. Market integration is increasing, as are interactions with loggers, merchants and colonists. Most Tsimane continue to fish, practice horticulture, hunt and gather for the majority of their subsistence. The demographic sample used here is based on reproductive histories collected by Gurven (*Gurven et al., 2017*) of 348 adults in 12 remote communities during 2002-2003. Changes in mortality are evident over the past ten years, and so mortality data used here are restricted to the years 1950-1989. Age estimation of older individuals was done by a combination of written records of missionaries, relative age rankings, and by photo and verbal comparison with individuals of known ages.

The **Gainj** are swidden horticulturalists of sweet potato, yams and taro in the central highland forests of northern Papua New Guinea. Meat is fairly rare (*Johnson, 1981*). At the time of study by Patricia Johnson and James Wood (1978-79 and 1982-83), there were roughly 1,318 Gainj living in twenty communities. Contact was fairly recent, in 1953 with formal pacification in 1963, and there is genetic and linguistic evidence of their relative isolation (Wood et al., 1982). Prior to contact, population growth had been zero for at least four generations (*Wood and Smouse, 1982*). An A2 Hong Kong influenza epidemic reduced the population by 6.5% in 1969-70, and probably accounts for the dearth of older people in this population. Data were obtained from government censuses from 1970-77, include non-Gainj Kalam speakers, and it is likely that ages are fraught with error for older adults ([*Wood and Smouse, 1982*](#_ENREF_17)). Additionally, published mortality estimates were already fitted with a Brass two-parameter logit model.

**Other populations**

We also add the Northern Territory Aborigines of Australia, an acculturated group of hunter-gatherers, and the pastoralist Herero of Botswana and Namibia.

The **Northern Territory Australian Aborigine** mortality data come from analysis of vital registration from 1958-1960 by Lancaster Jones (*Lancaster Jones, 1963, 1965*). At this time, few Aborigines in the region were still full time foragers. There was a significant amount of age-clumping at five year intervals, and so a smoothing procedure was done on the age distribution of the population. It is likely that infant deaths and more remote-living individuals are under-renumerated, and Lancaster Jones made adjustments to impute missing deaths. We view these data with caution but include them because no other reliable data exist for Australia, apart from a Tiwi sample culled from the same author.

The **Herero** are Bantu-speaking pastoralists studied by Renee Pennington and Henry Harpending from 1987-1989 (*Pennington and Harpending, 1991*). They are traditionally cattle and goat herders in the Kalahari Desert of the Ngamiland District of northwestern Botswana, numbering 10-15,000 during time of study. They had migrated to this area in the early 20^th^ century, due to displacements from the Herero-German War. They live in extended family homesteads without running water or electricity, remain endogamous, and are now very successful cattle herders. They also raise more drought-resistant goats and other livestock. Total fertility rates increased from 2.7 in the first half of the 20^th^ century to 7 in the 1980s; the lower earlier fertility was likely due to pelvic inflammatory disease stemming from sexually transmitted infections (*Gurven and Kaplan, 2007*).

For more details on these study populations, including methodological information on demographic samples, age estimates and mortality, see (Gurven and Kaplan 2007).

**2. Mortality smoothed with a Siler model**

We smooth survivorship (*l_x_*) and mortality (*h_x_*) functions using a Siler competing hazards model (*Siler, 1979; Gage, 1989*). The Siler model includes three components: a negative Gompertz exponential function to capture infant and juvenile mortality (*a_1_*, *b_1_*), a Makeham constant hazard (*a_2_*), and a positive Gompertz function to capture late age mortality (*a_3_*, *b_3_*). The hazard has the following functional form: $h(x)=a_{1}e^{-b_{1}x}+a_{2}+a_{3}e^{b_{3}x}.$We produce Siler-based *l_x_* and *h_x_* curves using the nonlinear regression procedure (NLIN) in SAS version 9.3.

**3. Fertility smoothed with local polynomial regression**

Age-specific fertility rates (*ASFR*) describe the average number of (age 0) newborns produced by an individual age *x*. For five of the chimpanzee groups (Gambia, Kanyawara, Ngogo, Taï and Taronga) and for seven of the human populations (Aborigines, Agta, Gainj, Herero, Hiwi, Ju/’hoansi !Kung and Yanomamo), *ASFR* are organized into 5-year age categories (where *ASFR* describes the average annual birth rate within each 5-year interval)(Note that we do not have population-specific fertility data for Ngogo chimpanzees so we use data from nearby Kanyawara). *ASFR* of the other four populations (Ache, Gombe chimpanzees, Hadza, and Tsimane) are reported in 1-year age intervals. For each of the 17 populations we use a local polynomial regression (loess, span = 0.5) to smooth noisy source data and interpolate annual age-specific fertility rates. The resulting *ASFR* are rescaled evenly such that their sum conserves the original *TFR*$\left(ASFR^{(final)}=ASFR^{(smoothed)}\left(TFR^{(raw)}/TFR^{(smoothed)}\right)\right).$

**4. Matrix model construction and emergent life history traits**

Using mean annual fertility rates (*m_x_*) and survival probabilities (*p_x_*) of individuals at each age *x* (indexed *i* or *j*)*,* we assemble a Leslie (*Leslie, 1945*) population projection matrix **A** (**A** = {*a_ij_*})(*Caswell, 2001*), where matrix elements *a_ij_* describe the number of age *i* individuals added to the population at time *t*+1 by one age *j* individual alive at time *t* (see Table 1 for variable definitions, source equations and details of matrix models). After correcting for sex ratio at birth (*SRB*, assumed here to be 1.05 live male newborns per live female newborn), the first row of the Leslie matrix **A** describes the average number of female newborns (age 0) birthed by mothers age *x* (*a_1x_* = *m_x_* = *ASFR* / (1+ *SRB*)); the first subdiagonal contains the probabilities of an individual surviving from age *x* to age *x*+1 (*a_x+1,x_* = *p_x_*). Reproductive maturity (menarche or estrus) is assumed at the minimum age (*α*) at first birth (*α* = min(*x*│*m_x_* > 0) and post-reproductive lifespan begins at the first age beyond the maximum age (*ω*) at last birth (*ω* = max(*x*│*m_x_* > 0) Aggregate population dynamics described by the matrix **A** reflect the additive contributions of individuals at every age and project forward the distribution **n***_x,t_* of individuals at each age *x* and at each time *t* (**n***_x_*,*_t_*_+1_ = **A n***_x_*,*_t_*). If vital rates don’t change then the population structure is stable, the population grows at the asymptotic growth rate *λ* (**n***_x_*,*_t_*_+1_ = *λ***n***_x,t_*)(*Caswell, 2001*), the stable age distribution is given by the right eigenvector **w,** and the reproductive values (*RV_x_*) of different ages (*x*) are given by the left eigenvector **v**. Population growth is also described by the intrinsic growth rate *r* (*r* = log *λ*), which is the annual (proportional) change in the population size.

For each age *x*, mortality rates (*μ_x_*) are calculated as the log of annual mortality probabilities *q_x_* (*μ_x_* = log (*q_x_*) = log (1-*p_x_*)) (fig. S3). Survivorship (*l_x_*) is the cumulative product of annual survival probabilities up to age *x* $\left(l_{x}=\prod_{a=0}^{x-1}(p_{a})\right)$; we are especially interested in survival to reproductive maturity at age *α* (*l_α_*), to the mean age at childbearing *MAC* (*l_M_*, see below), and to the maximum age of reproduction *ω* (*l_ω_*). Life expectancies (*e_0_*) are the sum of survivorship across age$\left(e_{0}=\sum_{x}(l_{x})\right)$and reflect the average age of death of individuals. The total fertility rate (*TFR*) is the sum of age-specific fertility rates$\left(TFR=\sum_{x}(ASFR)=\sum_{x}(2.05m_{x})\right)$and predicts completed fertility of individuals that survive beyond childbearing ages. Mean age of childbearing (*MAC*) is the average age of mothers in a population$\left(MAC=(2.05/TFR)\sum_{x}xm_{x}\right)$ . Mean age at first birth (*AFB*) and mean age at last birth (*ALB*) are calculated from the serial probabilities of parity progression up to the maximum age of death *T*$\left(AFB=\sum_{x}xm_{x}\prod_{a=0}^{x-1}(1-m_{a})\right)$;$\left(ALB=\sum_{x}xm_{x}\prod_{a=x}^{T}(1-m_{a})\right).$Inter-birth intervals (*IBI*) assume lifetime fertility (*TFR*) is spread evenly across the mean reproductive lifespan (*IBI* = (*ALB* – *AFB*) / *TFR*). Variable Definitions and source equations appear in Table 1.

**5. Force of selection: vital rate sensitivities and elasticities**

Vital rate elasticities *e_ij_* (*e_ij_* = (d λ / λ) / (d *a_ij_* / *a_ij_*)) are calculated analytically from the matrix **A** using published Matlab code (*Caswell, 2001*), and predict the change in the population growth rate (*λ*) caused by a proportional, infinitesimal perturbation of a matrix element (*a_ij_*), each containing either a fertility rate (*a_1x_*  = *m_x_*) or survival probability (*a_x+1,x_* = *p_x_*). These equations utilize the eigenvectors (**v** and **w**) of the population projection matrix **A**, representing the stable age (*i*) distribution (**v** = {*v_i_*}) and the reproductive value of age *j* individuals (**w** = {*w_j_*}) in the population, to estimate matrix element sensitivities *s_ij_*( (*s_ij_* = *v_i_w_j_* / < **w**,**v** >). We use these sensitivities to estimate elasticities *E_ij_* (*E_ij_* = (*a_ij_* / *λ*) *s_ij_*), which predict the proportional change in the population growth rate (*λ*) caused by a proportional change in a vital rate (*p_x_* or *m_x_*) . Because elasticities conveniently sum to unity across all vital rates (1 = **Σ***_ij_* *E_ij_*), we can also estimate the total elasticity of survival$\left( E_{s}=\sum_{x} E_{x+1,x} \right)$, or fertility$\left( E_{f}=\sum_{x} E_{1x};1=E_{s}+E_{f} \right)$, or sum elasticities across certain age ranges to get total (composite) elasticity to child survival or adult survival$\left( E_{c}={E_{c}=\sum_{x<\alpha} E_{x+1,x} ;E_{a}=\sum_{x\geq\alpha} E_{x+1,x} ; E_{f}=\sum_{x} E_{1x} ;}E_{s}=E_{c}+E_{a} \right)$. In every case maximum sensitivities elasticities are to infant survival (max(*E_ij_*) = *E_21_* = *s_21_* (*a_21_* / *λ*)). Following Mesterton-Gibbons (2000) we impute sensitivities of fertilty as zero for all ages outside the observed range of nonzero fertility and elasticities are zero outside this range because they scaled by mean vital rates.

**6. Life table response experiments (LTRE): vital rate fitness contributions**

Differences (Δ*λ*, Δ*r*) in population growth rates (*λ*, *r* = ln(*λ*)) are decomposed into positive and negative contributions to the total difference in *λ* using a one-way fixed-treatment life table response experiment (LTRE; *Caswell, 1989, 2001*). We compare the ten human populations (fig. S5) in our sample to a reference life history (**A**^(HG)^) exhibiting the mean fertility and survival rates estimated for hunter-gatherers $\left(\mathbf{A}^{(\text{HG})}=(1/N_{HG})\sum_{m=1}^{N_{HG }}\mathbf{A}^{(m)}\right),$where *m* is the population index (*m* = 1, 2, 3, …, *M*) and *N_HG_* is the number of hunter-gatherer societies (*N_HG_* = 5). We also compare each of seven chimpanzee populations to this (averaged) synthetic reference (HG) (fig. S6). By comparing each population to a common reference, contributions standardize the population growth effects of vital rate differences. Positive contributions increase the population growth rate and negative contributions decrease the population growth rate of each population *n*, relative to the hunter-gatherer reference (HG). We also estimate contributions for synthetic populations with vital rates equal to the average across non-exclusive foragers$\left(\mathbf{A}^{(NF)}=(1/N_{NF})\sum_{m=1}^{N_{NF}}\mathbf{A}^{(m)}\right),$where *N_NF_* is the number of non-forager populations (*N_NF_* = 5), or across synthetic populations with vital rates averaged across declining wild chimpanzee populations or across increasing wild chimpanzee populations (fig. 3*A,B*).

Contributions *C_ij_* are the product of the difference (Δ*a_ij_*) in a matrix element (*a_ij_*) and the sensitivity (*s_ij_*) of the population growth rate to that element. Differences in each vital rate are calculated between each target population (*m*) and the reference life history (HG)$\left(\Delta a_{ij}=a_{ij}^{(m)}-a_{ij}^{(\text{HG})}\right)$. Contributions *C_ij_* (*C_ij_* = *s_ij_*Δ*a_ij_*) sum to estimate the total difference in population growth rate$\left(\Delta\lambda=\lambda^{(m)}-\lambda^{(\text{HG})}\approx\sum_{i,j}C_{ij}\right)$, so the population growth rates of each target population *λ*(*^n^*) are predicted by adding the sum of all contributions to the reference (HG) population growth rate $\left(\lambda^{(m)}\approx\lambda^{(\text{R})}+\sum_{i,j}C_{ij}\right)$. We also calculate the relative *effects* of vital rates, estimated as the proportion$(C_{ij*})$of the combined effects of all contributions that are attributed to each vital rate (*p_x_*, *m_x_*), the magnitudes of which sum to unity across vital rates and are thus directly comparable to elasticities$\left(C_{ij*}=\left|C_{ij}\right|\div\sum_{i,j}\left|C_{ij}\right|;\right.$ $\left.1=\sum_{i,j}C_{ij*}\right).$We are particularly interested in the relative effects of fertility (*C_f_*) vs. survival (*C_s_*) $\left(C_{s}=\sum_{x=0}^{T}\left|C_{x+1,x}\right|\div\left(\sum_{x=0}^{T}\left|C_{x+1,x}\right|+\sum_{x=\alpha}^{\omega}\left|C_{1x}\right|\right)\right.;$ $C_{f}=\sum_{x=\alpha}^{\omega}\left|C_{1x}\right|\div\left(\sum_{x=0}^{T}\left|C_{x+1,x}\right|+\sum_{x=\alpha}^{\omega}\left|C_{1x}\right|\right);$ $\left.1=C_{s}+C_{f}\right),$the proportion (*C_c_*) of effects due child survival vs. adult survival $\left(C_{c}=\sum_{x=0}^{\alpha-1}\left|C_{x+1,x}\right|\div\left(\sum_{x=0}^{T}\left|C_{x+1,x}\right|+\sum_{x=\alpha}^{x=\alpha}\left|C_{1x}\right|\right);\right.$ $C_{a}=\sum_{x=\alpha}^{T}\left|C_{x+1,x}\right|\div\left(\sum_{x=0}^{T}\left|C_{x+1,x}\right|+\sum_{x=\alpha}^{x=\alpha}\left|C_{1x}\right|\right);$ $\left.C_{s}=C_{c}+C_{a}\right)$and degree to which the potential effects suggested by elasticities are realized in vital rate contributions. The last of these are reflected in the scalars *Z_c_*, *Z_a_* and *Z_f_*, which are the ratios of the combined effect to the total elasticity of child survival (*Z_c_* = *C_c_* : *E_c_*), adult survival (*Z_a_* = *C_a_*: *E_a_*), or fertility (*Z_f_* = *C_f_* : *E_f_*) (Table 2).

LTRE predictions rely on vital rate sensitivities (*s_ij_*) to scale the effects of differences in vital rates (Δ*a_ij_*), so the sensitivities we use affect the accuracy of our predictions (*Benton and Grant, 1999*). Because they usually generate more accurate prediction (*Logofet and Lesnaya, 1997*), we use sensitivities of a hypothetical ‘midpoint’ projection matrix (**A**^(M)^), whose entries are the average of the target population (**A**(*^m^*)) and the hunter-gatherer reference (**A**^(HG)^)$\left(\mathbf{A}^{\text{(M)}}=\{a_{ij}^{\text{(M)}}\};a_{ij}^{\text{(M)}}=0.5\left(a_{ij}^{\text{(}m\text{)}}+a_{ij}^{(\text{HG})}\right)\right).$ Sensitivities $\left(s_{ij}^{\text{(M)}}\right)$of the midpoint matrix (**A**^(M)^) also lie midway between the sensitivities of the target$\left(s_{ij}^{(m)}\right)$and reference$\left(s_{ij}^{(\text{HG})}\right)$, and are nonzero when matrix elements (*a_ij_*) are nonzero in either the target or reference population. Thus, any vital rate that differs between populations and is nonzero in either the target or reference population contributes to the overall difference (Δ*λ*) in the population growth rates (Δ*λ* = *λ*(*^m^*)- *λ*^(HG)^).

**Supporting Information for Davison and Gurven “**Human uniqueness? Life history diversity among small-scale societies and chimpanzees**”.**

**S2 File: Supporting Results**

**1. Population growth and decline**

Among small-scale societies, the Tsimane have the highest population growth rate (*r* = 3.8%), followed by the Yanomamo (*r* = 3.3%), Ache (*r* = 2.6%), Aborigines (*r* = 1.7%), and the Hadza (*r* = 1.4%) (figs. 3, 4; Table 2). The Agta (*r* = 0.5%), Gainj (*r* = 0.3%), Hiwi (*r* = 0.04%) and Ju/’hoansi !Kung (*r* = 0.07%) exhibit near-zero growth. Chimpanzee populations are growing at Ngogo (*r* = 3.3% assuming Kanyawara fertility) and at Kanyawara (*r* = 0.94%) but declining at Mahale (*r* = ‑0.39%) and Gombe (*r* = ‑1.36%) and crashing at Taï (*r* = ‑9.6%). The reintroduced population in Gambia is growing slowly (*r* = 0.49%) and the Taronga Zoo population is growing (*r* = 4.1%).

**2. Mortality and fertility patterns**

The lowest infant (age 0 to 1) mortality in our sample is among the protected Gambia chimpanzees and the lowest mortality between ages 2 and 4 is among the Ngogo chimpanzees. The highest mortality above age 1 is also among chimpanzees (Mahale between ages 1 and 3, 3 to 43 at Taï, 43 to 53 at Gombe, and over 53 at Mahale). Among small-scale societies, mortality rankings are not consistent across the life cycle. The Ache have the lowest mortality in the first year of life (age 0 to 1), the Yanomamo between ages 1 and 3, acculturated Aborigines between ages 3 and 43, and the Herero above age 43 (fig. S3). The highest human mortality in our sample is among the Agta for infants (age 0 to 2) and between ages 25 and 43, the Ju/’hoansi between ages 2 and 4, the Hiwi between 4 and 24, the Gainj between 43 and 67, and the Hiwi beyond age 67.

Among chimpanzees, Gambia has the lowest mortality of newborns (age 0 to 1) and after age 60, Ngogo has the lowest mortality between ages 1 and 10 and between ages 28 and 60, Mahale between ages 10 and 28. Among chimpanzeees, infant mortality (ages 0 to 3) and mortality between over age 43 is highest at at Mahale, and highest between ages 3 and 43 at Taï (fig. S3).

Among the small-scale societies in our sample, fertility is highest before age 15 and after age 40 among the Ache, between ages 15 and 25 among the Yanomamo and between ages 25 to 30 among the Agta. While reproduction begins before age 15 in some (Aborigines, Ache, Hadza, Tsimane), the Gainj and the Ju\’hoansi !Kung do not reproduce until age 20. The lowest nonzero fertility between ages 10 and 15 is found among the Hadza, between ages 15 and 20 among the Hiwi, between ages 20 and 25 among the Gainj, and after age 25 among the Herero.

Chimpanzees in the Taronga Zoo begin reproducing at low rates as early as age 5 but the other populations don’t start until age 10. Chimpanzee fertility estimates are extremely variable across age and across populations, partly due to small sample sizes (*Thompson et al., 2007*).

**Supporting Information for Davison and Gurven “**Human uniqueness? Life history diversity among small-scale societies and chimpanzees**”**

**S3 File: Supporting References**

Benton, T., and A. Grant. 1999. Elasticity analysis as an important tool in evolutionary and population ecology. Trends in Ecology and Evolution 14:467-471.

Blurton Jones, N. 2016. Demography and Evolutionary Ecology of Hadza Hunter-Gatherers. Vol. 71. Cambridge University Press, Cambridge, UK.

Blurton Jones, N. G., K. Hawkes, and J. F. O'Connell. 2002. Antiquity of postreproductive life: Are there moodern impacts on hunter-gatherer postreproductive life spans? American Journal of Human Biology 14:184-205.

Blurton Jones, N. G., J. F. O'Connell, K. Hawkes, C. L. Kamuzora, and L. C. Smith. 1992. Demography of the Hadza, an increasing and high density population of savanna foragers. American Journal of Physical Anthropology 89:159-181.

Caswell, H. 1989. Analysis of life table response experiments I. Decomposition of effects on population growth rate. Ecological Modelling 46:221-237.

Caswell, H. 1996. Second derivatives of population growth rate: calculation and applications. Ecology 77:870-879.

Caswell, H. 2000. Prospective and retrospective perturbation analyses: their roles in conservation biology. Ecology 81:619-627

Caswell, H. 2010. Life table response experiment analysis of the stochastic growth rate. Journal of Ecology 98:324-333.

Chagnon, N. 1968. Yanomamo: the Fierce People. Holt, Rinehart and Winston.

Davison, R., H. Jacquemyn, D. Adriaens, O. Honnay, H. de Kroon, and S. Tuljapurkar. 2010. Demographic effects of extreme weather events on a short‐lived calcareous grassland species: stochastic life table response experiments. Journal of Ecology 98:255-267.

Davison, R., Nicolè, F., Jacquemyn, H. & Tuljapurkar, S. 2013. Contributions of covariance: decomposing the components of stochastic population growth in Cypripedium calceolus. American Naturalist 181:410-420.

Early, J. D., and T. N. Headland. 1998. Population dynamics of a Philippine rain forest people: The San Ildefonso Agta. University Press of Florida, Gainsville, FL.

Early, J. D., and J. F. Peters. 2000. The Xilixana Yanomami of the Amazon: History, Social Structure, and Population Dynamics. University Press of Florida, Gainsville, FL.

Gage, T. 1989. Bio-mathematical approaches to the study of human variation in mortality. Yearbook of Physical Anthropology 32:185-214.

Fisher, R. A. 1930. The genetical theory of natural selection. Dover, NY

Grant, A. 1997. Selection pressures on vital rates in density–dependent populations. Proceeding of the Royal Society of London: Biological Sciences 264:303-306.

Gurven, M. & Kaplan, H. 2007. Longevity among hunter-gatherers: a cross-cultural comparison. Population and Development Review 33:321-365.

Gurven, M., J. Stieglitz, B. Trumble, A. D. Blackwell, B. Beheim, H. Davis, P. Hooper, and H. Kaplan. 2017. The Tsimane health and life history project: Integrating anthropology and biomedicine. Evolutionary Anthropology: Issues, News, and Reviews 26:54-73.

Headland, T. N. 1997. Revisionism in ecological anthropology. Current anthropology 38:605-630.

Hernández‐Pacheco, R. R. G. Rawlins, M. J. Kessler, L. E. Williams, T. M. Ruiz-Maldonado, J. González‐Martínez, A. V. Ruiz-Lambides, and A. M. Sabat. 2013. Demographic variability and density‐dependent dynamics of a free‐ranging rhesus macaque population. American Journal of Primatology 75:1152-1164.

Hill, K. R., and A. M. Hurtado. 2017. Ache Life History: The Ecology and Demography of a Foraging People. Routledge.

Hill, K., A. M. Hurtado, and R. S. Walker. 2007. High adult mortality among Hiwi hunter-gatherers: implications for human evolution. Journal of Human Evolution 52:443-454.

Horvitz, C., D. Schemske, and H. Caswell. 1997. Pages 247-271 *in* Tuljapurkar, S, and H. Caswell, eds. Structured-population models in marine, terrestrial, and freshwater systems. Springer, US.

Howell, N. 1979. Demography of the Dobe !Kung. Academic Press, New York, NY.

Howell, N. 2010. Life histories of the Dobe! Kung: food, fatness, and well-being over the life span. Vol. 4 Univ of California Press.Hurtado, A. M. & Hill, K. R. 1987. Early Dry Season Subsistence Ecology of the Cuiva Foragers of Venezuela. Human Ecology 15:163-187.

Hurtado, A. M., and K. Hill. 1990. Seasonality in a foraging society: Variation in diet, work effort, fertility, and the sexual division of labor among the Hiwi of Venezuela. Journal of Anthropological Research 46:293-34.

Johnson, P. L. 1981. When dying is better than living : female suicide among the Gainj of Papua New Guinea. Ethnology 20:325-334.

Jones J. 2009. The force of selection on the human life cycle. Evolution and Human Behavior 30:305-314.

Kaplan, H., K. Hill, J. B Lan.caster and A. M. Hurtado. 2000. A theory of human life history evolution: Diet, intelligence, and longevity. Evolutionary Anthropology 9:156-185.Lancaster Jones, F. 1963. A Demographic Survey of the Aboriginal Population of the Northern Territory, with Special Reference to Bathurst Island Mission. Australian Institute of Aboriginal Studies.

Lancaster Jones, F. 1965. The Demography of the Australian Aborigines. International Social Science Journal 17:232-245.

Logofet, D., and E. Lesnaya. 1997. Why are the middle points the most ‘sensitive’ in the sensitivity experiments? Ecological Modelling 104:303-306.

Mesterton-Gibbons, M. 2000. A consistent equation for ecological sensitivity in matrix population analysis. Trends in Ecology & Evolution 15:115.

Morris, W. F., J. Altmann, D. K. Brockman, M. Cords, L. M. Fedigan, A. E. Pusey, T. S. Stoinski, A. M. Bronikowski, S. C. Alberts, and K. B. Strier. 2010. Low demographic variability in wild primate populations: fitness impacts of variation, covariation, and serial correlation in vital rates.  American Naturalist 177:E14-E28.

Muller, M., and R. Wrangham. 2014. Mortality rates among Kanyawara chimpanzees. Journal of Human Evolution 66:107-114.

Pennington, R. & Harpending, H. 1991. Infertility in Herero pastoralists of southern Africa. American Journal of Human Biology 3:135-153.

Pennington, R., and H. Harpending. 1993. The structure of an African pastoralist community: demography history and ecology of the Ngamiland Herero. Clarendon Press.

Siler, W. 1979. A competing-risk model for animal mortality. Ecology 60:750-757.

Solway, J. S., and R. B. Lee. 1990. Foragers, genuine or spurious?: situating the Kalahari San in history. Current Anthropology 31:109-146.

Thompson, M., J. H. Jones, A. E. Pusey, S. Brewer-Marsden, J. Goodall, D. Marsden, T. Matsuzawa, T. Nishida, V. Reynolds, Y. Sugiyama, and R. W. Wrangham. 2007. Aging and fertility patterns in wild chimpanzees provide insights into the evolution of menopause. Current Biology 17:2150-2156.

van Tienderen, P. 2000. Elasticities and the link between demographic and evolutionary dynamics. Ecology 81:666-679.

Tuljapurkar, S. 2013. Population dynamics in variable environments. Vol. 85. Springer Science and Business Media.

Wisdom, M. J., L. S. Mills, and D. F. Doak. 2000. Life stage simulation analysis: estimating vital rate effects on population growth for conservation. Ecology 81:628-641.

Wood, B., D. Watts, J. Mitani, and K. Langergraber. 2017. Favorable ecological circumstances promote life expectancy in chimpanzees similar to that of human hunter-gatherers. Journal of Human Evolution 105:41-56.

Wood, J. W., and P. E. Smouse. 1982. A method of analyzing density-dependent vital rates with an application to the Gainj of Papua New Guinea. American Journal of Physical Anthropology 58:403-411.

Wood, J. W., P. L. Johnson, R. L,. Kirk, K. McLoughline, N. M. Blake and F. A. Matheson. 1982. The genetic demography of the Gainj of Papua New Guinea. I. Local differentiation of blood group, red cell enzyme, and serum protein allele frequencies. American Journal of Physical Anthropology 57:15-25.

**Supporting Tables for Davison and Gurven “**Human uniqueness? Life history diversity among small-scale societies and chimpanzees**”**

**S1 Table. Study populations and metadata.** Human populations are arranged by subsistence type (H: hunter-gatherers, A: acculturated hunter-gatherers, F: foraging-horticulturalists, P: pastoralists) and chimpanzees are arranged into wild (W), managed (M) and captive (C) populations. Columns contain information on location, region or continent, habitat type, population size, study period and data sources for fertility and mortality rates.

† Composite populations: Ngogo analyses use fertility data from nearby Kanyawara and Taronga analyses use mortality data averaged over three zoo populations.

**S2 Table. Age-specific survival probabilities (*p_x_*) for study populations.** Survival probabilities are used as single-age survival transitions in matrix **A** (*p_x_* = *a_x+1,x_*; **A** = {*a_ij_*}), and constructed from hazard rates smoothed with a Siler model.

S2 table is uploaded separately as an Excel File.

**S3 Table. Age-specific fertility rates (*m_x_*) for study populations**. Fertility rates are used as single-age contributions of neonates (*n_0_*) in the matrix **A** (*m_x_* = *a_1x_* = *ASFR*/*SRB*; **A** = {*a_ij_*}), assuming *SRB* = 1.05. *ASFRs* are smoothed using a Loess regression.

S3 table is uploaded separately as an Excel File.

**S4 Table. Mann-Whitney-Cox *p*-values and *U*-statistics for differences in mean values between humans and wild chimpanzee populations.** For tests of differences in mean population growth rate (*r* = log *λ*), mortality measures, fertility measures, or selection measures (units above first row of values), rows show difference tests (*p*, *U*) for all humans vs. chimpanzees (*H.s*. vs. *P.t.*), for hunter-gatherers vs. chimpanzees (HG vs. *P.t*.), for non-foragers vs. chimpanzees (NF vs. *P.t.*), and for hunter-gatherers vs. non-foragers (HG vs. NF) Lower rows show means (x̄) and standard deviations (SD) of each measure hunter-gatherers (HG), non-foragers (NF) or all humans (*H.s.*) and across declining (WC-) or increasing populations (WC+) of wild chimpanzees or across all wild chimpanzees (*P.t*.). NS results are indicated by greyed cells (*p* > 0.1) and marginal significance (*p* < 0.1) by lightly greyed cells; bold values indicate comparisons in which a given measure is significantly higher among humans (or hunter-gatherers) vs chimpanzees or among hunter-gatherers vs. non-foragers. Note that statistics (*x̄* ± SD) are calculated across populations in each grouping and may differ from life history traits calculated from mean (composite) life histories.

**S5 Table. Mann-Whitney-Cox *p*-values and *U*-statistics for differences in mean values between different contribution measures for a given life history.** Rows show Mann-Whitney-Cox *U*-statistics and *p*-values for differences in mean values between five hunter-gatherer societies (HG, n = 5), five non-exclusive forager societies (NF, n = 5), all ten human populations (*H.s.*, n = 10), or five wild chimpanzee populations (*P.t*., n = 5). Columns to the left show results for tests of differences in either the total effect (combined magnitude of contributions) made by pairwise differences between total survival (*C_s_*), child survival (*C_c_*), adult survival (*C_a_*) and fertility (*C_f_*), or for differences in the relative accuracy of elasticities in predicting the fitness importance of child survival (*Z_c_*) vs. either adult survival (*Z_a_*) or fertility (*Z_f_*). Columns to the right report means (*x̄*) and standard deviations (SD) for each of these composite measures across a given set of populations. NS results are indicated by greyed cells (*p* > 0.1) and marginal significance (*p* < 0.1) by lightly greyed cells; bold values indicate comparisons in which the first in a given pair of measures is significantly higher. Note that statistics (*x̄* ± SD) are calculated across populations in each grouping and may differ from life history traits calculated from mean (composite) life histories.

**Supporting Figures for Davison and Gurven “**Human uniqueness? Life history diversity among small-scale societies and chimpanzees**”**

**S1 Fig. Fertility smoothing comparison (raw vs. smoothed).** Fertility smoothed with a local polynomial regression (loess; span 0.5, blue lines) is compared with raw *ASFR* from source literature. (O) Note that for the Hadza, published rates are already smooth.**
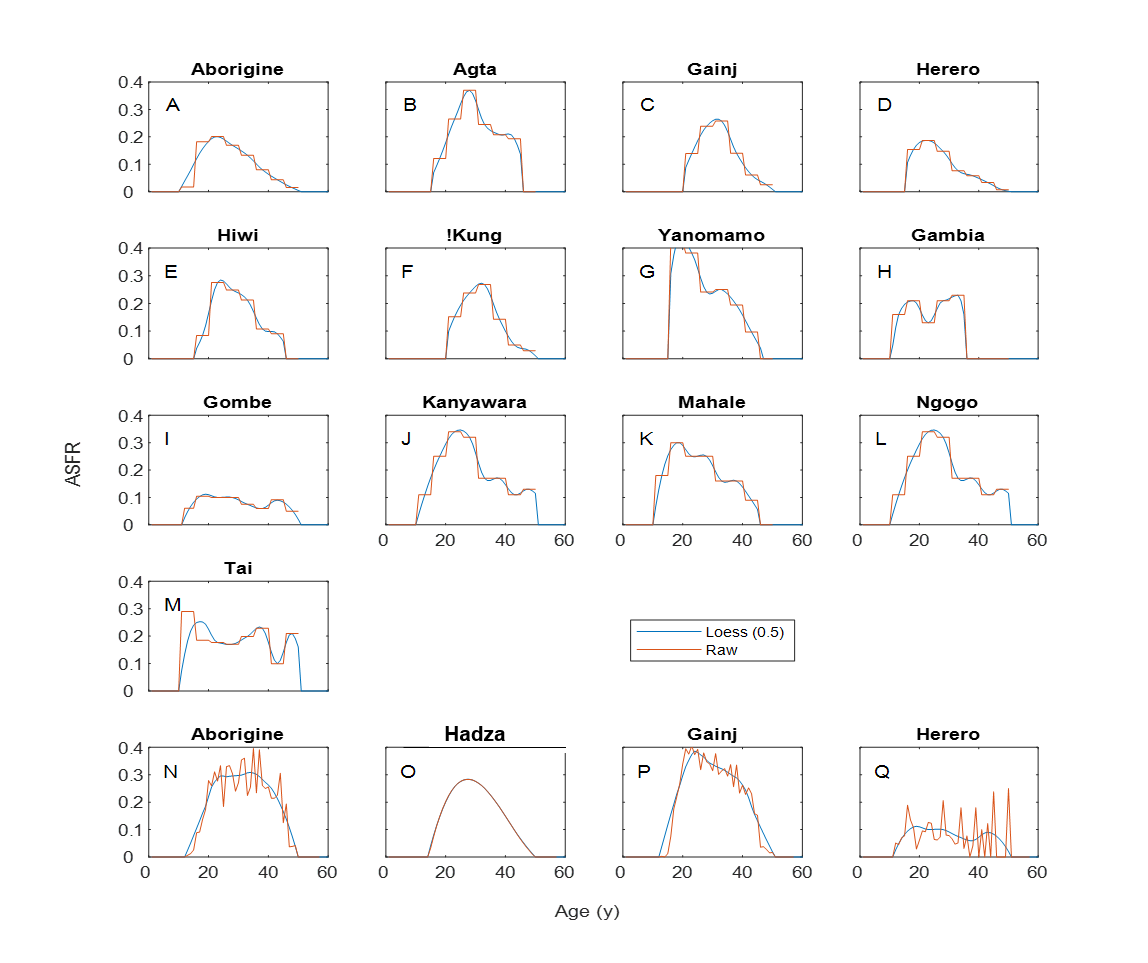
**

**S2 Fig**. **Fertility tempo comparisions.** (A), Estimated mean ages at first birth (*AFB*) are compared with literature values. (B), Esimated age at last birth (*ALB*). Our estimates using fertility (*ASFR*) and survivorship (*l_x_*) are on the *x*-axes and literature values (sources listed in S1 Table) are on the *y*-axes. Dashed lines show 1:1 parity line, solid lines show significant linear regressions and inset text reports correlation coefficients (*r^2^*) and significance *p*-values.


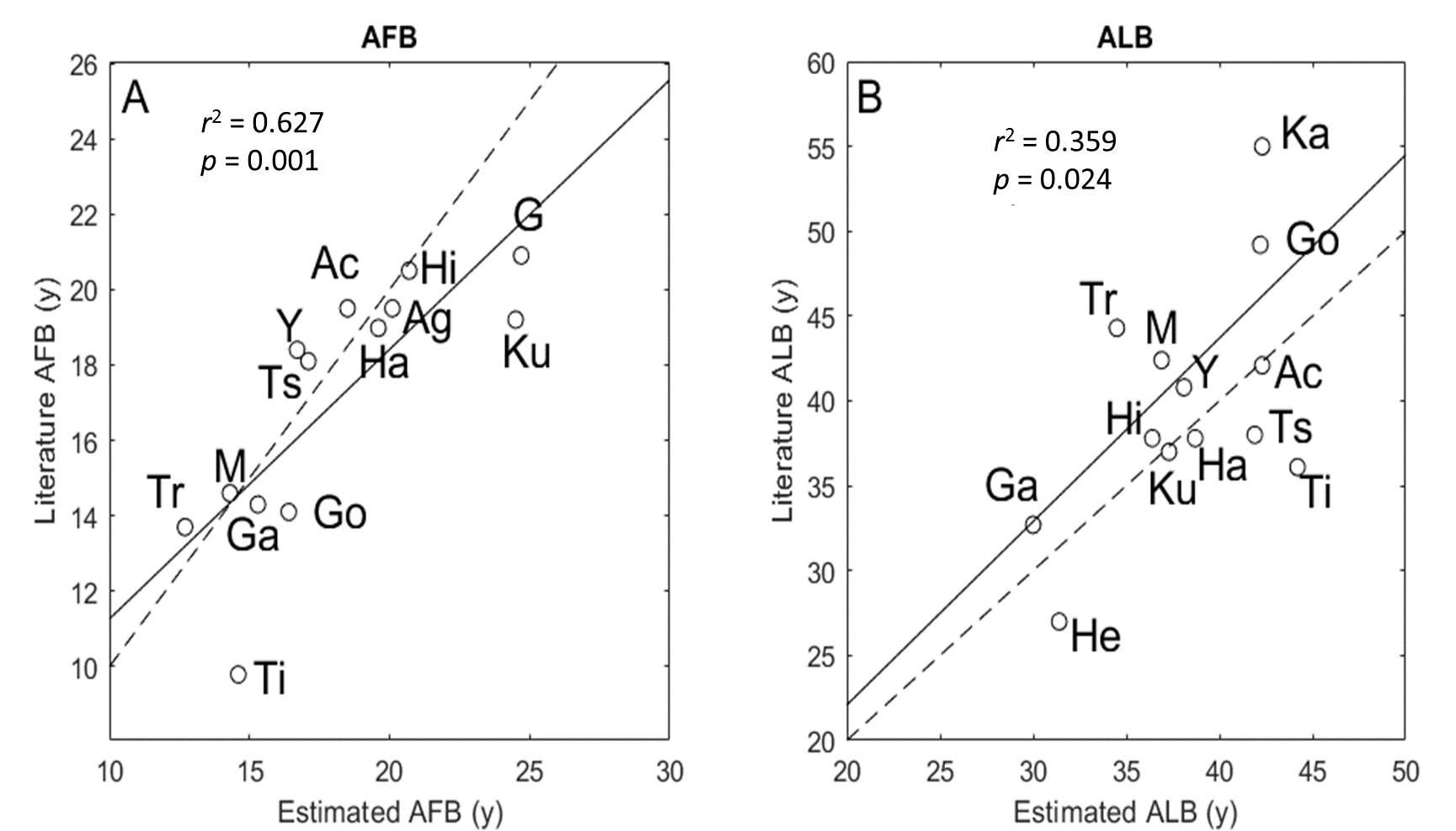


**S3 Fig.** **Mortality and fertility rates for individual populations.** Mortality *μ_x_* (blue lines, left *y*-axes) and fertility *ASFR* (red lines, right *y*-axes) are shown for each age *x* (*x*-axes). Black dots indicate neonate mortality (*μ_0_*). Vital rates are estimated for ten small-scale human subsistence societies and seven chimpanzee populations (parenthetical labels indicate ecology type as in S1 Table). (T-W) Bottom panels show results for the mean life histories calculated across hunter gatherers (T), non-foragers (U), wild chimpanzees (V), declining chimpanzees (O), increasing chimpanzees (S), or all human small-scale societies (W).

† Composite populations with mean rates: (O) declining P.t. mean, (S) increasing *P.t*. mean, (T*)* hunter-gatherer mean, (U), non-forager mean, (V) *P.t*. mean, (W) *H.s.* Mean), (*N*) Ngogo assuming fertility from nearby Kanyawara, (*H*) Taronga uses pooled zoo mortality.

**S4 Fig. Vital rate elasticities.** Elasticity to survival (*E_s_*, blue lines, left *y*-axes) and to fertility (*E_f_*, red lines, right *y*-axes) are shown for each age *x* (*x*-axes). Elasticities are estimated for ten small-scale human subsistence societies and seven chimpanzee populations (parenthetical labels indicate ecology type as in S1 Table). (T-W) Bottom panels show results for the mean life histories calculated across hunter gatherers (T), non-foragers (U), wild chimpanzees (V), or across all human small-scale societies (W); Bottome right panels show mean life histories calculated for declining chimpanzees (O), increasing chimpanzees (S).

† Composite populations with mean rates (HG Mean, NF Mean, *H.s*. Mean, WC- Mean, WC+ Mean, *P.t.* Mean), or in the case of Ngogo, fertility from nearby Kanyawara, and in the case of Taronga using pooled zoo mortality.

**S5 Fig.** **LTRE contributions among small-scale societies.** Using average vital rates of hunter-gatherers as a reference, differences in population growth rate (*λ*) are decomposed into contributions from survival (*C_s_*) and fertility (*C_f_*) for ten small-scale human socieities, arranged by subsistence type (as in Table 2, S1 Table). (C), inset text reports negative neonate (age 0) survival (*p_0_*) contribution exceeding the *y*-axis limits.

**S6 Fig.** **LTRE contributions among chimpanzees.** Using average vital rates of hunter-gatherers as a reference, differences in population growth rate (*λ*) are decomposed into contributions from survival (*C_s_*) and fertility (*C_f_*) for seven populations of chimpanzees, including five wild populations (labeled W), one managed population founded by released captives (labeled M) and one captive population (labeled C). (D), inset values show negative contributions of neonate (age 0) survival (*p_0_*) and infant survival (*p_1_*) that exceed axis limits.

† Results for Ngogo use fertility data from nearby Kanyawara and results for Taronga Zoo use mortality averaged across three zoo populations

**S7 Fig. Summed contributions for each population.** Stacked bars show summed contributions of infant, child and adult survival and of early, prime and late fertility. Positive and negative contributions are summed separately to reflect the net difference in population growth rate (white bars), relative to the composite mean hunter-gatherer reference. The black-and-white line crossing the bars indicates the population growth rate (*r* = log(*λ*)). Results are shown for ten small-scale societies and five chimpanzee populations (labeled as in Figure 4).

**S8 Fig.** **Total effects of vital rate differences.** Total effects (**Σ** *C_ij_**) reflecting the combined magnitude of contributions within each population (labeled as in Figure 4, arranged in increasing order of population growth rate as in Figure S7). Stacked bars decompose the total effect (the proportion of the combined magnitude of all contributions) made by infant, child and adult survival and by early, prime and late fertility (inset text shows the percent of total effects).
