# Supplementary material for: Human uniqueness? Life history diversity among small-scale societies and chimpanzees
Source: PLoS One. 2021 Feb 22;16(2):e0239170. doi: 10.1371/journal.pone.0239170 (PMC7899333; doi:10.1371/journal.pone.0239170)
Supplement: Attachment — Submitted filename: PlusOne_D_20_26560_review.pdf [file pone.0239170.s002.pdf]

Authors of this manuscript showed that delayed maturity and adult mortality is the main difference to separate humans from chimpanzees which shares common ancestor. They employed Life Table Response Experiments to quantify vital rate contributions to population growth rate. Their results and discussion are is interesting. Their approach is justified. However, this manuscript contains numerous inconsistencies in its data and statements. It requires careful attention on details to justify its results and conclusions.

Major inconsistencies and questions:

- Lines 213 - 227, Table 1: In the manuscript, line 180 states that $Z_a = C_a/E_a$ and lines 148 and 150 shows that $E_c + E_a + E_f = 1$. However, numbers in the table are inconsistent to the statement in text. $Z_a \neq C_a/E_a$ and $E_c + E_a + E_f \neq 1$
- Lines 213 - 227, Table 1: Why $E_0 = E_f$?
- Lines 213 - 227, Table 1: Why do managed and captive population have smaller ALB compare to Wild population for chimpanzees?
- Fig 3A: Why are there two yellow and red sections in the bar of WC- and WC? Why are there two red and brown sections in the bar of WC+? Why does Infant survival (the purple section) have both positive and negative contributions in WC- and WC?
- Lines 337-338: "$E_0 > E_c > E_a > E_f$" is not consistent with numbers in Table 1.
- Line 345: "$C_c > C_a$" is not consistent with numbers in Table 1.
- Line 350: "$C_s \approx C_f$" is not consistent with numbers in Table 1.
- Lines 357-359: "$Z_c \approx Z_a$", "$Z_c < Z_a$" and "$Z_c < Z_a$" are not consistent with numbers in Table 1.

Minor points:

- Line 127: please spell out "NLIN".
- Lines 131 to 133: Is it a duplicated statement?
- Line 146: "$(e_{ij} = (\partial\lambda/\partial a_{ij}) = s_{ij}(\lambda/a_{ij})$" should be "$(e_{ij} = (\partial\lambda/\partial a_{ij}) = s_{ij}(a_{ij}/\lambda))$".
- Line 153: "$\sum_{i,j} s_j \Delta a_{ij}$" should be "$\sum_{i,j} s_{ij} \Delta a_{ij}$".
- Line 184: "$\max_{i,j}(e_{ij})$" should be '$\max(e_{ij})$".
- Table 1 row 1: Please add description of l_α, l_M, l_ω, TFR, AFB, MAC, ALB and IBI in the caption.
- Table 1 row 1: Please switch column C_a and C_c for display consistency.

- Line 278, Fig 2: Cannot find the “non-foragers by filled squares” in Fig 2.
- Line 279, Fig 2: Cannot find the “(labeled NF)” in Fig 2.
- Line 349: “S4 Table” should be ”S2 Table”.
- Line 352: “S4 Table” should be ”S2 Table”.
- Line 360: “S4 Table” should be ”S2 Table”.
- It will be helpful to add a data table of computed age specific mortality and fertility of each population (which are the data used to plot Fig 1) into online supplementary.
