# Supplementary material for: Human uniqueness? Life history diversity among small-scale societies and chimpanzees
Source: PLoS One. 2021 Feb 22;16(2):e0239170. doi: 10.1371/journal.pone.0239170 (PMC7899333; doi:10.1371/journal.pone.0239170)
Supplement: Attachment — Submitted filename: Reviewe_PlosOne_2020.pdf [file pone.0239170.s003.pdf]

Review of ***Human uniqueness illustrated by life history diversity among small-scale societies and chimpanzees*** by Raziel Davison and Michael Gurven.

In this article, the authors compare age-trajectories of survival and fertility of many hunter-gatherer and forager human populations and chimpanzees. They then perform LTRE and spectral analysis to investigate potential evolutionary changes between these trajectories. This is a very interesting manuscript, revisiting with originality and up-to-date data a classic question in human evolutionary biology: the evolution of the human and chimpanzee life-cycle since divergence. The manuscript incorporates a wonderful comparison of age-trajectories of the different not-industralized populations with several chimpanzee populations, again using the best data to date. In this respect I think that this article has great potential. I am more sceptical about the evolutionary interpretation of the LTRE and elasticity analysis. I think that there are several conceptual and technical issues which need to be addressed prior to publication.

Main comments 1

The authors mainly use two metrics in the analyses: elasticities and what they called *fitness contributions*. All over the manuscript, I found unclear the definition of *fitness contribution,* and what the two measures together brings to the analyse.

First, I think there is a problem of definition throughout the manuscript. This starts L 52-55 where I found the sentence "*These fitness contributions illustrate how life history event schedules drive differences within and between species, while elasticities reflect the force of selection and highlight the potential for fitness contributions if vital rates vary across populations [19]*" not very clear (illustrate? drive difference of what on what?). The authors quote [19] which focuses (to my knowledge) exclusively on elasticity and tells anything (as far as I remember it) on contrasting elasticity and 'fitness contribution'.

The authors are later more explicit when referring to LTRE where they are defined as the "*vital rate contributions to observed differences in population growth rates*" between two projection matrices (please note that vital rates are not individual measures as mentioned in l71 since there are population aggregates). In this sense, they are not "*contribution to* [a population] *fitness*" but how differences in entries of two matrices translate into difference in change of population reference growth rate. I strongly suggest the authors to define it more clearly. A way to do it is that sensitivity s_{ij} is the impact on λ of one unit of change in matrix entry a_{ij}. If we multiply s_{ij} by $\Delta a_{ij,}$ it tells us how such a change would have modify the reference population growth λ.

Second, the authors then states l74-75 that "*Differences between realized fitness contributions and the potential suggested by elasticities may indicate constraints on life history evolution*" (also 405-406). This can be a fantastic idea and I can intuit what the authors have in mind. Yet it is not trivial to me, and it makes me wonder if this has been already theorized elsewhere. If it has, the author should clearly state it and explain why (I think not shying away equations). If it has not, I would strongly encourage the authors to develop - and if possible demonstrate - this idea. For instance, C_{ij}, is a given amount of change between two matrix waited by sensitivities. Does this idea relates to the long lasting debate on the difference between using sensitivities and elasticities?

Main comment 2

The authors used the ratio between contribution and elasticity to measure (if I understand well) these possible constraints. But, I would strongly suggest the authors to check the resulting equation. First, l146, I think there is a mistake: e_{ij} is not equal to $s_{ij}*(\lambda/a_{ij})$ but to $s_{ij}*(a_{ij}/\lambda)$ (I guess that this is a typo because elasticities look ok in fig 1) .

But then $Z_{ij} = C_{ij}/E_{ij} = (\Delta a_{ij}.s_{ij})/(s_{ij}(a_{ij}/\lambda))=(\Delta a_{ij}/ a_{ij})(1/\lambda)$.

Then Z is the 'percentage' of difference between the reference and the analysed matrices divided by the growth rate. I am far on being clear on what does this mean and how this allows identifying constraint on a

vital rate. I therefore strongly suggest the authors to explicit this metric and how/why it is used to solve their research question.

I am also not clear on whether Z should be sum(C_{ij})/sum(E_{ij}) or rather sum(C_{ij}/E_{ij}), which can be substantially different.

Finally I don' t understand the values for the Zs in table 1. For instance for Ache, Zc=Cc/Ec=7/42=0.16, not 95. Or, am I missing something?

I would suggest to incorporate Table S5 into the main text.

Main comment 3

I am not sure that I understand prediction 1 and it may be there a conceptual mistake. Canalization is the fact that vital rates impacting the more fitness (here λ) should exhibit lower **temporal variance** than those under weaker selection. The authors rightfully quote [22] and [23] testing this by somehow correlating the estimation of the variation in time of matrix entries to the variation of λ (but variance is in time, not between populations, isn't it?).

Note also that, if I am not mistaken, [23] performed elasticity analysis not LTRE (as suggested in sentence l84) such that the effect of variance on LTRE is also not that clear to me. Anyway, I cannot see how LTRE between populations (without temporal variance accounted for) can allow identifying life-history constraint and how the concept of canalization is involved into this. If I am mistaken, I strongly suggest the authors to make their point more clear.

Main comment 4

I find that P1 (l81-82) is not well formulated. If I am not mistaken, it is a property of elasticity to be strictly declining with age in an age-structured model, infant and children survival elasticity always being constant and the largest. Metric have to be twisted and parameters very different that those of mammals to find alternative pattern (Baudish, 2005, PNAS). It is between species that relative magnitude of elasticities can be compared and I would strongly suggest to cite Heppell et al., 2000, Ecology for a comparison in mammals across the slow-fast continuum. Also why not refering to and using a classical Silvertown triangle to represented this (Silvertown, J., et al. 1993. Jouranl of Ecology 81:465–476)?

I am not sure what the authors want to test with prediction 2 which is the obvious fact that both increasing survival and fertility should increase population growth rate. Evidencing trade-off between fertility and survival?

Main comment 5

I would suggest the authors to discuss limitations of elasticities analyses in general and apply to humans in particular. (1) First elasticities are only one hand of the evolutionary GxE equation (Lande 1982; Charlesworth 1990; Steppan et al. 2002). Evolution also need genetic variance and this could be acknowledged. (2) The authors are comparing Leslie matrix, but any sub structuring (as individual heterogeneity) or hidden trade-offs may change the results. (3) It the most important, it has been shown that intergenerational transfers between age-class or parental investment can strongly impacts elasticities on survival and fertility in humans (Lee 2003, PNAS, Pavard et al. 2007, Evolution, Pavard & Branger 2012 Theo Pop Biol). For instance, magnitude of elasticities on adult survival may be strongly underestimated when maternal or grand-maternal care is not implemented. Elasticities on fertilities by age can also exhibit very different patterns. Because such intergenerational transfers have been proposed as a very important drivers of the evolution of human life-history, the authors should at least discuss it. (4) As the authors wonderfully argued in a recent article, only periodic catastrophes in humans can explain the human forager

paradox. It also means that all elasticity analysis in constant and infinite environment is somehow incomplete and elasticity should be considered into a stochastic model.

Minor comments & Détails

l418-420 – Isn't there a contradiction is stating that juvenile survival is under canalization effect and stating later on that it varies more in time than adult survival?

Figure 1 – I am not sure how the SEM of elasticity is calculated. Is this trivial?

I am not sure what figure 2 really brings to the article since it is complicated and barely discussed.

Problem in legend of figure 2 – Non-Forager are filled-circled as HG not filled square. Indicate that isolines are population growth rates. Remove the title.

L290 – I guess this is fig3.B instead of 2B,3?

l 32 – I am not sure that reference [2] did anything in calculating the divergence time between humans and chimpanzees. Please check carefully this reference. I think it should instead be referred to l 33-34.

l 35, "*human fertility is similar to chimpanzees*" and further. Please be more specific. Do you mean the shape of the age-specific fertilities? If yes, both the distribution and the TFR? Is the whole shape the distribution identical? The authors refer to [6] who focus mainly on reproductive senescence and show that if the timing of reproductive senescence is similar rate of reproductive senescence is not the same as well as how it correlates with decline in survival. I suggest to be more precise.

L37-38 – "*However, there is great variation among human and chimpanzee life histories*". Here again I suggest to be more specific. The authors quote [8]. Although a valid reference, it can be completed by more recent article (as the [2]). Furthermore, I am not a native speaker but is "difference" would be better than "variation"?

L39 and many time after – Please change "within species" by between population. In ecology, within species study refers more to the study of variance between individuals than between population as it is investigate here.

L 40 - "*We interpret population life histories in terms of the slow-fast life history continuum [9]*" – Why? Also, human a complete outlier on this continuum so that I wonder if this is relevant.

L41 – To my knowledge Stearns' book (but I don't have it at hand here to check) is about trade-offs in general not about their importance for human life-history evolution.

In [6] the authors use extensive data in chimpanzees. Yet, this represent only about 600-1000 individuals (the equivalent of a small human village) spread in small groups over nearly a continent. How this could affect the authors' results?

L55 – I am not found of the concept of population fitness underlying in this sentence.

L71 – Indicates the pages in [15]. Note that you could have also quoted Hal Caswell, 1989, Analysis of life table response experiments I. Decomposition of effects on population growth rate, Ecological Modelling, Volume 46, Issues 3–4.

L153 – this should be sij instead of sj isn't it?

L179 – I am not sure to understand why the fact that Cij and Eij sum to unity allow to calculate the ratio.

Figure 3A – I find the figure very complicated to figure out. Are they the mean summed C values between populations? Then why and how is separated positive and negative C values? Or "composite" refers to the

mean trajectories for HG, F, WC. But then, again, how does it lead to both (+) and (-) for a same trait (i.e., Infant survival). I am very sorry if I miss this information.
